# Targeted IFNγ induction by a genetically engineered *Salmonella typhimurium* is the key to the liver metastasis inhibition in a mouse model of pancreatic neuroendocrine tumor

**DOI:** 10.3389/fmed.2023.1284120

**Published:** 2023-10-31

**Authors:** Zhan Hua, Shan Wu, Yulian Zhang, Xiuhong Wang, Ju Cui, Yunxuan Li, Chengcheng Yang, Min Zhai, Bo Deng, Bin Yu, Jian-Dong Huang, Zai Wang, Jianjun Zhou

**Affiliations:** ^1^Department of General Surgery, Institute of Clinical Medical Sciences, China-Japan Friendship Hospital, Beijing, China; ^2^Research Center for Translational Medicine, Cancer Stem Cell Institute, East Hospital, Tongji University School of Medicine, Shanghai, China; ^3^Department of Neurosurgery, Institute of Clinical Medical Sciences, China-Japan Friendship Hospital, Beijing, China; ^4^Department of Pathology, China-Japan Friendship Hospital, Beijing, China; ^5^The Key Laboratory of Geriatrics, Beijing Institute of Geriatrics, Beijing Hospital, National Center of Gerontology, National Health Commission, Institute of Geriatric Medicine, Chinese Academy of Medical Sciences, Beijing, China; ^6^NHC Key Laboratory of Biotechnology of Antibiotics, Institute of Medicinal Biotechnology, Chinese Academy of Medical Sciences & Peking Union Medical College, Beijing, China; ^7^Ningbo First Hospital, Ningbo, China; ^8^Institute of Clinical Medical Sciences, China-Japan Friendship Hospital, Beijing, China; ^9^School of Biomedical Sciences, Li Ka Shing Faculty of Medicine, The University of Hong Kong, Hong Kong, Hong Kong SAR, China; ^10^HKND YB1 Pharmaceutical Limited, Hong Kong, Hong Kong SAR, China

**Keywords:** *Salmonella typhimurium*, YB1, liver metastasis, pancreatic neuroendocrine tumor, targeted IFNγ therapy

## Abstract

**Background:**

Liver metastasis is one of the primary causes of death for the patients with pancreatic neuroendocrine tumors (PNETs). However, no curative therapy has been developed so far.

**Methods:**

The anti-tumor efficacy of a genetically engineered tumor-targeting *Salmonella typhimurium* YB1 was evaluated on a non-functional INR1G9 liver metastasis model. Differential inflammatory factors were screened by Cytometric Bead Array. Antibody depletion assay and liver-targeted AAV2/8 expression vector were used for functional evaluation of the differential inflammatory factors.

**Results:**

We demonstrated that YB1 showed significant anti-tumor efficacy as a monotherapy. Since YB1 cannot infect INR1G9 cells, its anti-tumor effect was possibly due to the modulation of the tumor immune microenvironment. Two inflammatory factors IFNγ and CCL2 were elevated in the liver after YB1 administration, but only IFNγ was found to be responsible for the anti-tumor effect. Liver-targeted expression of IFNγ caused the activation of macrophages and NK cells, and reproduced the therapeutic effect of YB1 on liver metastasis.

**Conclusion:**

We demonstrated that YB1 may exhibit anti-tumor effect mainly based on IFNγ induction. Targeted IFNγ therapy can replace YB1 for treating liver metastasis of PNETs.

## Introduction

Pancreatic neuroendocrine tumors (PNETs) account for about1-2% of pancreas-originated tumors (1, 2). Except for small insulinoma, all other PNETs should be considered potentially malignant, although the malignancy is much lower than pancreatic ductal adenocarcinomas (1, 3). While functional PNETs cause a serious of clinical symptoms such as hypoglycemia, diarrhea, gastrointestinal ulcer, etc., which drive the patients to clinical attention at an earlier stage; non-functional PNETs (NF-PNETs) grow asymptomatically. About 40% of all patients were incidentally diagnosed (4, 5), and liver metastases were already present in about half of the patients at first visit (1, 2), which has a worse outcome than local or regional tumors, with a median survival duration of about only 2 years (1). Therefore, the development of treatments for unresectable or metastatic PNETs, especially NF-PNETs, is urgently needed.

Immune therapy is one of the leading anti-tumor strategies. It was originally established by Doctor William B Coley as bacterial-mediated anti-tumor therapy (6), developed into diverse mode of immune cell-mediated therapy nowadays. Recently, genetically engineered bacteria were created as new anti-tumor bullets. Salmonella is a facultatively anaerobic bacterium which has brilliant anti-tumor ability. In order to eliminate its toxicity, several attenuated strain such as VNP20009 (7), A1-R (8) and YB1 (9) have been artificially engineered or screened out as potential anti-tumor drugs.

YB1 was designed as an obligate anaerobic salmonella strain. It was first demonstrated to be effective in treating breast cancer and later neuroblastoma (10). It enriched in the necrotic region of the tumor and recruited neutrophil granulocyte to kill the tumor cells. However, it was discovered later that YB1 also functioned well in tumors without obvious necrosis region, such as hepatic cell carcinoma (11). This made it possible that YB1 may have additional anti-tumor mechanisms such as blood vessel targeting, which has been reported in other salmonella strains. In addition, it has been shown that YB1 administration can induce IFNγ secretion and activate NK cells to eliminate tumor cells. However, it is questionable whether IFNγ can be used as a monotherapy to treat tumor metastasis, considering that systemic infusion of IFNγ could not inhibit the tumor metastasis to the lung (12).

Since it is relatively dangerous to apply live bacteria in the clinical treatment of cancer, the clear understanding of the underlined mechanism and the development of the replacement therapy is of significant importance. In this study, we demonstrated that YB1 could inhibit the liver metastasis in a PNET mouse model through IFNγ, and liver-targeted expression of IFNγ could reproduce the anti-metastasis efficacy of YB1 and hold great potential as a new therapeutic strategy for treating liver metastasis of tumors.

## Materials and methods

### Cell culture

Gold hamster INR1G9 cells (13) were cultured in RPMI 1640 (GIBCO, US) medium (11.2 mM glucose) supplemented with 5% fetal bovine serum and 10^5^ U/L penicillin and 100 mg/L streptomycin. 293 T cells (ATCC) were cultured in high glucose DMEM (GIBCO, US) medium supplemented with 10% fetal bovine serum.

### Bacteria culture

Bacterial strain YB1was kindly provided by Dr. JD Huang (HKU, China). YB1 was grown in LB medium, with supplements of 25 μg/mL Chloramphenicol, 50 μg/mL Streptomycin and 100 μg/mL 2,6-Diaminopimelic acid (DAP) (Sigma, US), with shaking at 220 rpm over night at 37°C. The concentration of the overnight culture was determined by plating with series of dilution.

### *In vitro* infection assay

INR1G9 cells were seeded at 2.5 × 10^4^/well in 24-well plate 24 h before infection. 5 × 10^6^ YB1 from overnight culture were centrifuged and resuspended in culture medium for INR1G9 cells. Then the bacteria and INR1G9 cells were co-cultured for 2 h. INR1G9 cells were washed with D-Hanks and further cultured in 50 μg/mL Gentamycin supplemented medium to remove the extracellular bacteria for another 24 h.

### Xenograft tumor models

The Animal Ethics Committee of China–Japan Friendship Hospital reviewed and approved all animal experiments (No. 180210), which were performed according to the Principles of Laboratory Animal Care. 6-8-week-old female nude (nu/nu) mice from Charles River Laboratories, Inc. were used in this study. Mice were maintained under specific-pathogen-free conditions and had access to food and water *ad libitum*. The feeding conditions were as follows: 24 ± 2°C; 50 ± 10% relative humidity; 12 h light/dark cycle. Mice were acclimatized to the laboratory conditions for 5–7 days prior to experimentation. The animal protocol was designed to minimize pain or discomfort to the animals. For intra-spleen inoculation, the mice were anesthetized with an intraperitoneal injection of 1% pentobarbital sodium (45 mg/kg). The skin was disinfected with 75% alcohol and an oblique incision was made on left side to pull out the spleen. 1 × 10^6^ INR1G9 cells in 25 μL FBS-free culture medium were injected into the spleen using BD insulin syringe^®^ until visible splenic capsule swelling. After injection and needle withdrawal, dry cotton swab was used for hemostasis and cell leakage oppression. Then the spleen was replaced into the abdominal cavity and the skin was sutured (14). Since the growth of INR1G9 tumor did not cause obvious body weight loss and changes of physical conditions, no euthanasia was carried out before the planned end of the experiment. At the end of the experiment, the animals were e euthanized by the intraperitoneal injection of 0.1 mL of 200 mg/mL pentobarbitone sodium.

### *In vivo* infection by YB1

5 × 10^7^ bacteria were harvested from overnight culture, centrifuged, resuspended in 100 μL PBS, and injected into the tail vein of the mice (*n* = 10) one week after INR1G9 inoculation. The mice were kept for another 3 weeks and then euthanized to harvest the tumors. For the antibody neutralizing assay, rat anti-IFNγ or anti-CCL2 antibodies (Bioxcell, US) (2 mg/kg) were injected intraperitoneally twice a week for three weeks after YB1 treatment.

### Immunohistochemistry, immunofluorescence, and antibodies

For histological analysis, INR1G9 tumors were fixed in 4% PFA/PBS overnight, and then prepared into paraffin sections for H&E staining. For immunohistochemistry, the deparaffinized sections were pretreated with 10 mM sodium citrate buffer for antigen unmasking, blocked in 3% H_2_O_2_ followed by normal serum, incubated with rabbit anti-salmonella (Abcam, UK) at 4°C overnight. Sections were incubated with HRP-conjugated goat anti-rabbit antibody (DAKO, Denmark) at room temperature for 1 h. Diaminobenzidine tetrachloride was used for color development, and the slides were counterstained with hematoxylin. For immunofluorescence on cultured cells, the cells were fixed with 4% PFA/PBS for 10 min followed by permeabilization using 0.2% Triton X-100/PBS for 10 min before staining. For immunofluorescence on tissues sections, the livers of the mice (*n* = 3) injected with AAV vectors were fixed in 4% PFA/PBS for 2 h, balanced in 30% sucrose/PBS overnight, and then prepared into frozen sections before staining. Immunofluorescent staining was performed according to the standard procedure. The primary antibodies used were: mouse anti-iNOS (Abcam, UK), mouse anti-NK1.1 (Novus, CH), rat anti-F4/80 (R&D, US). The secondary antibodies used were Alex Fluor^®^ 488 Donkey Anti-Rabbit IgG (H + L), Alex Fluor^®^ 555 Donkey Anti-Rat IgG (H + L) and Alex Fluor^®^ 555 Donkey Anti-Mouse IgG (H + L) (Invitrogen, US). DAPI was used to stain the cell nuclei.

### Detection of inflammatory factors in the liver

A small piece of mouse liver (*n* = 5) was taken, weighed, homogenized in PBS with 0.1% Triton X-100 and protease inhibitors (Roche, Switzerland) and centrifuged to remove the precipitations. The inflammatory factors including IL-12, p70, TNF, IFNγ, CCL2, IL-10, IL-6 were detected using Cytometric Bead Array (CBA) Mouse Inflammation Kit (BD, US). The supernatant was incubated with the capture beads and the detector reagents in one tube. The fluorescent intensity in APC channel was used to discriminate the capture beads and the PE intensity was used to detect cytokine levels. The CBA assay was performed on BD FACSCantoplus™. To measure IFNγ alone in the mice liver injected with AAV vectors through the tail vein (*n* = 3), IFN γ Factor ELISA Kit (Beijing 4A Biotech, China) was used according to the instructions.

### Construction of AAV2/8 packaging plasmids

AAVpro^®^ Helper free system (AAV2) system was purchased from Takara, Japan. In order to construct liver specific promoter, two copies of ApoE enhancer followed by AAT promoter sequence (15) was synthesized and cloned into the HindIII/NruI sites in pAAV-CMV to replace the CMV promoter and generate pAAV-AAT. In order to track the distribution of AAV *in vivo*, the EGFP gene (sequence from pEGFP-C1, Clontech, US) was synthesized and constructed into the EcoRI/BamHI sites of pAAV-AAT to obtain pAAV-EGFP. To generate IFNγ-expression AAV vector, the mouse IFNG coding sequence (NM_008337.4) was synthesized and constructed into the EcoRI/BamHI site of pAAV-AAT to obtain pAAV-IFNr. In order to obtain AAV2/8 with liver specific infection, the VP1 gene of AAV8 was synthesized^1^ and cloned into SwaI/NdeI sites in pRC2-mi342 vector to replace the VP1 of AAV2 and generate pAAV2/8.

### Packaging of AAV2/8

In order to package chimeric AAV2/8, 6 × 10^6^ 293 T cells were inoculated into T75 flask 24 h in advance, and 9 μg pHelper, pAAV2/8 and AAV expression vectors (pAAV-AAT, pAAV-EGFP or pAAV-IFNr) were cotransfected into 293 T cells, and the solution was changed after 12 h. After 60 h, 15 mL culture supernatant was collected. Cells were digested and collected. Cell precipitates were resuspended with 3 mL lysis buffer (50 mm Tris, 150 mM NaCl, 2 mM MgCl_2_, pH 8.0) and freeze-thawed repeatedly for three times. The lysate was centrifuged at 3,000 rpm x 5 min to remove cell debris. Benzonase (100u/mL) was added and incubated at 37°C for 1 h. 15 mL supernatant, 3 mL cell lysate and 4.5 mL PEG8000 solution (40% PEG8000, 2.5 N NaCl) were mixed and ice bathed for 2 h, centrifugated for 2,500 g x 30 min at 4°C. The precipitation was resuspended with 13 mL DMEM, and passed through 0.45 μm filter to remove impurities. After ultracentrifugation for 150,000 g × 3 h at 4°C, the precipitation was resuspended with 2 mL serum-free 1,640 medium (16). AAV was titrated by real-time PCR using AAVpro titration kit (TaKaRa, Japan).

### *In vivo* treatment of AAV2/8

In order to detect the liver specific targeting of AAV2/8, 10^7^/100 μL AAV-EGFP virus was injected into the mice through tail veins. After 7 days, the heart, liver, pancreas, stomach, intestine and spleen were taken, and the green fluorescence signal in each organ was observed under fluorescence stereomicroscope (M205FA, Leica). In order to treat the tumor with AAV vector, 10^7^/100 μL AAV-IFNr or AAV vector AAV-AAT were injected into the tail vein of mice (*n* = 5) after INR1G9 cells were injected into the spleen. Four weeks later, the livers were harvested to detect the tumor growth.

### Statistical analysis

Images were processed and analyzed by ImageJ^®^. Student’s *t*-test was used to compare the differences between two groups. In case of more than two-group comparisons, one-way analysis of variance followed by the Tukey’s *post hoc* test was used. All statistical analyses were performed using the GraphPad Prism software version 7.04 (GraphPad Software, Inc.). *p < 0.05, **p < 0.01, ***p < 0.001 or ****p < 0.0001 was considered significant as indicated in the figure legends.

## Results

### YB1 cannot infect INR1G9 effectively *in vitro*

INR1G9 cells were isolated from gold hamster insulinoma cell In-111-R1 by single cell cloning and recently established as a non-functional PNET cell model (14). We tested whether YB1 could infect INR1G9 cells under normal culture condition. We also used hepatocellular carcinoma cell line Huh7.5 as a positive control. We found that the replication foci were present in a very small fraction of INR1G9 cells (Supplementary Figure S1A), while vast amount of YB1 were present in most of the Huh7.5 cells (Supplementary Figure S1B), showing that the infection of YB1 on INR1G9 cells was inefficient *in vitro*.

### Monotherapy of YB1 strongly inhibit INR1G9 liver metastasis without tumor cell targeting

We have demonstrated that while INS-1 can hardly form liver metastatic foci by intrasplenic injection, INR1G9 cells could be applied as an efficient liver metastasis model (14). The metastatic foci had a white-to-bloody appearance. The metastasis rate was 100% in less a month, and the number of metastatic foci reached to more than one hundred in about half of the mice.

**Figure 1:**
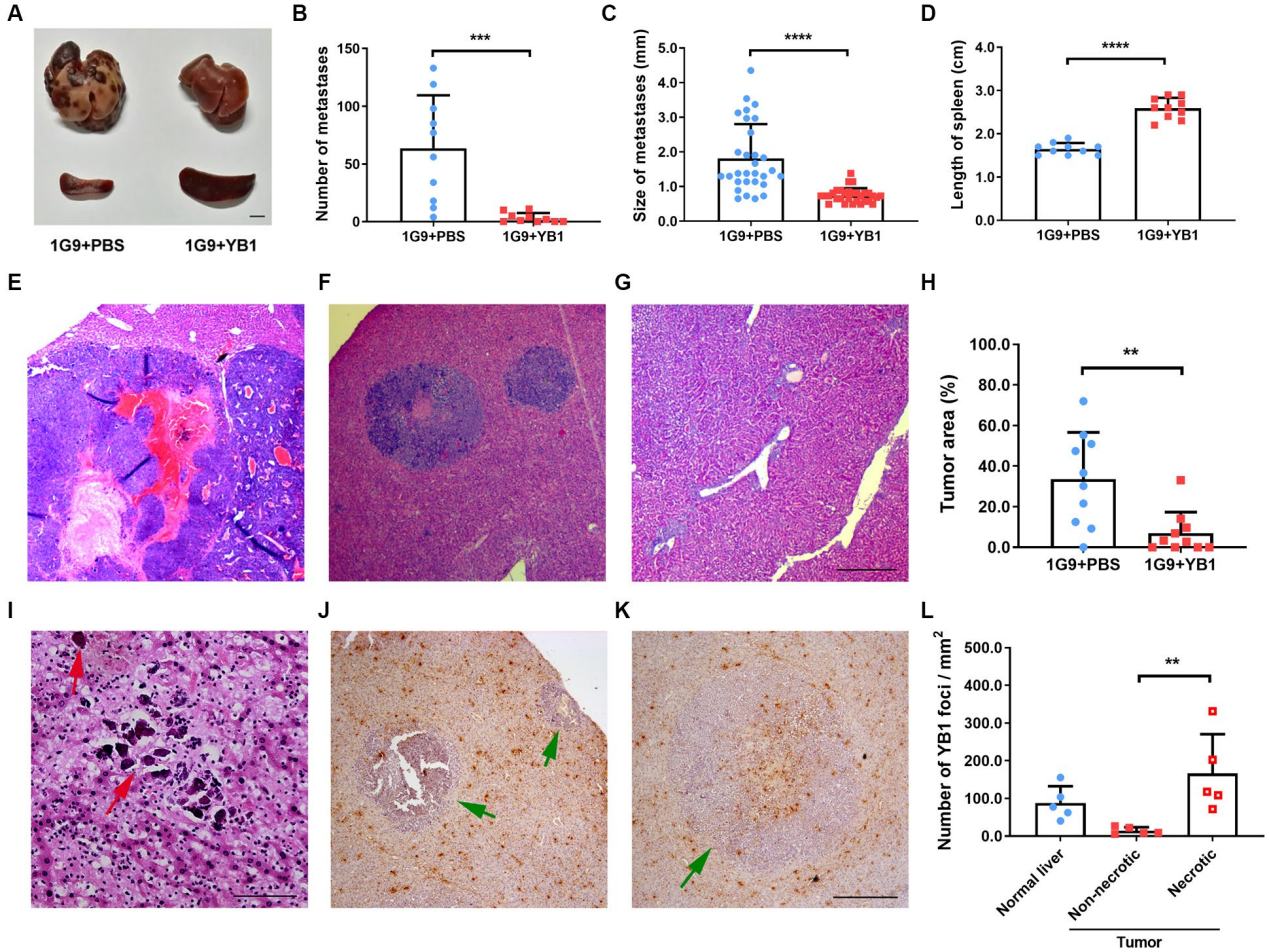
YB1 inhibited liver metastasis without infecting tumor cells in INR1G9 mouse models. **(A)** Representative pictures of the livers and spleens from mice inoculated with INR1G9 cells in the spleen and injected with PBS (1G9 + PBS) or YB1 (1G9 + YB1) through the tail veins (*n* = 10). Scale bar: 500 μm. **(B–D)** The number of liver metastases **(B)**, size of liver metastases **(C)** and the length of the spleen **(D)** was measured in the 1G9 + PBS and 1G9 + YB1 groups. **(E–G)** H&E staining of the liver sections of the mice inoculated with INR1G9 cells in the spleen and injected with PBS **(E)** or YB1 **(F,G)** through the tail veins. **(H)** Statistical analysis of the proportions of tumor area as represented in **(E–G)** (*n* = 10). **(I)** H&E staining of the liver sections of the mice inoculated with INR1G9 cells in the spleen and injected with YB1 through the tail veins. Red arrows: calcification foci. **(J,K)** The mice liver sections from the 1G9 + YB1 group were immunostained with anti-salmonella antibodies. Green arrows: INR1G9 metastatic tumors. **(L)** Quantitative analysis of YB1 foci in the liver or tumor areas as represented in **(J,K)**. Five immunostaining areas were randomly selected for normal liver, non-necrotic tumor and necrotic tumor, respectively. Scale bar in **(E–G)** and **(J,K)**: 500 μm; scale bar in **(I)**: 50 μm. Data are presented as mean ± SD. **p < 0.01; ***p < 0.001; ****p < 0.0001.

We tested the anti-tumor efficacy of YB1 on INR1G9 liver metastasis model. YB1 was administrated through tail vein one week after tumor implantation, and tumor growth was examined 3 weeks after treatment. As a result, YB1 strongly inhibited the liver metastases, and the color of the metastatic tumor turned much paler than those untreated (Figure 1A). The number of metastatic foci reduced remarkably (Figure 1B), and the size of the metastatic foci also decreased significantly (Figure 1C). At the same time, the size of spleen increased significantly as consequence of salmonella infection (Figure 1D). Four of ten liver even did not have obvious metastatic foci. The inhibition of liver metastases was verified by H&E staining (Figures 1E–H). We also observed calcification sites without any tumor cells around, suggesting that some metastatic tumor cells may be eliminated by the treatment (Figure 1I). When checking the distribution of the bacteria in the tumors, we found that they were rarely detected in the live tumor cells, but mainly in the necrotic tumor area and regions outside of the tumors (Figures 1J–L).

### IFNγ is necessary for efficient YB1 therapy

**Figure 2:**
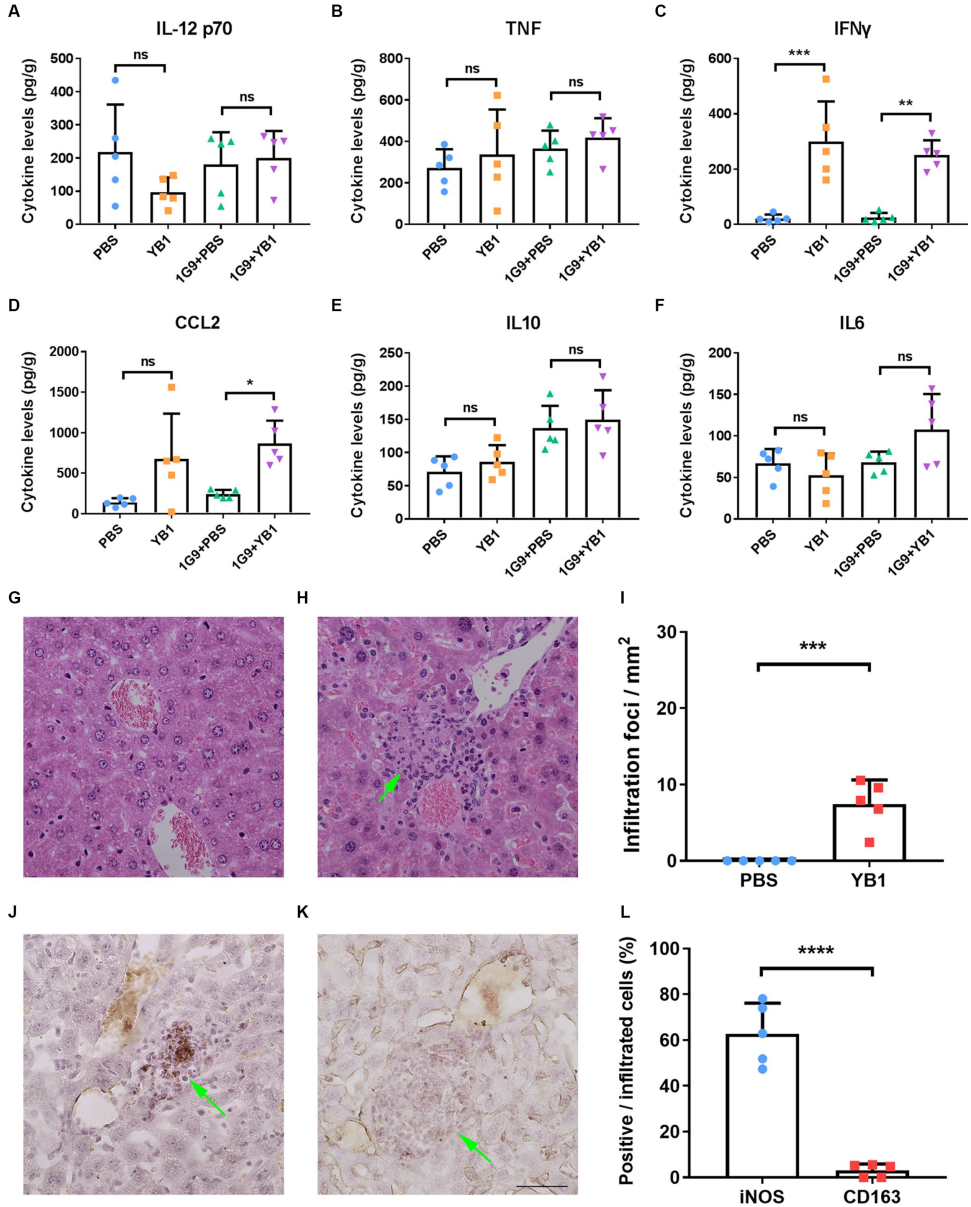
IFNγ and CCL2 levels were elevated in INR1G9 inoculated mice livers after YB1 treatment with M1 macrophages recruited. **(A–F)** Mice were injected with PBS or YB1 through the tail vein following intrasplenic injection of culture medium or INR1G9 cells (*n* = 5). The levels of inflammatory factors including IL12 p70 **(A)**, TNF **(B)**, IFNγ **(C)**, CCL2 **(D)**, IL10 **(E)**, and IL6 **(F)** in the mice livers were measured using cytometric bead array. **(G,H)** H&E staining of the liver sections from the mice injected with PBS **(G)** or YB1 **(H)** through the tail vein and kept for one week (*n* = 5). **(I)** Quantitative analysis of infiltration foci in the livers of PBS or YB1 injected mice as represented in **(G,H)** (*n* = 5). **(J,K)** Immunohistochemistry of the liver sections in **(I)** with anti-iNOS **(J)** or anti-CD163 antibodies **(K)** to show M1 or M2 macrophages, respectively. **(L)** Quantitative analysis of the percentages of immunostaining positive cells in the infiltrated cells as represented in **(J,K)** (*n* = 5). Data are presented as mean ± SD. Ns, non-significant; *p < 0.05; **p < 0.01; ***p < 0.001; ****p < 0.0001.

As previously reported that YB1 proliferates well in the liver (9), and bacterial infection may activate the immune system and promote the anti-tumor effect of the body, we speculate that the level of local inflammatory factors in the liver may change after YB1 infection, so as to activate the innate immune system. We took the liver of mice infected with YB1 for one week and detected IL-12, p70, TNFα, IFNγ, CCL2, IL-10 and IL-6 inflammatory factors. We found that the YB1 infected group had higher IFNγ and CCL2 than the uninfected group, while other inflammatory factors tested showed no significant difference (Figures 2A–F).

Since CCL2 functions in monocytes chemotaxis and IFNγ promote the M1 polarization of macrophages, we hypothesized that the upregulation of these two factors by YB1 may recruit and activate myeloid monocyte/macrophages. We observed clusters of infiltrating cells near the vasculature after YB1 injection (Figures 2G–I), and these cells were stained positive for M1 macrophage marker iNOS but not M2 marker CD163 (Figures 2J–L).

**Figure 3:**
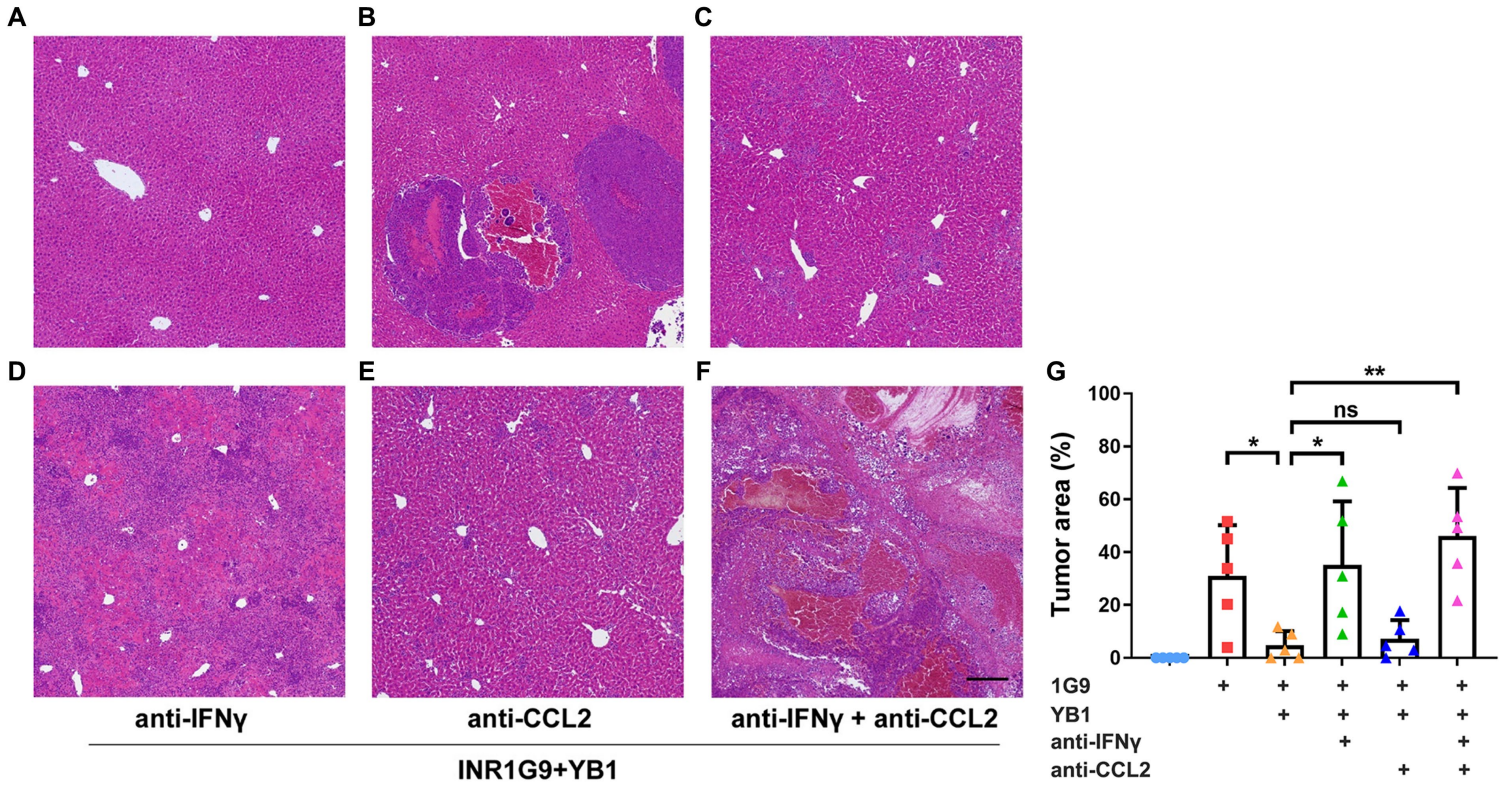
Anti-IFNγ but not anti-CCL2 abrogated the anti-tumoral effect of YB1. **(A–F)** H&E staining of the liver sections from the control mice **(A)** or mice with INR1G9 inoculated **(B)**, INR1G9 inoculated and treated with YB1 **(C)**, INR1G9 inoculated, treated with YB1 and anti-IFNγ **(D)**, anti-CCL2 **(E)** or anti-IFNγ plus anti-CCL2 **(F)**. Scale bar: 500 μm. **(G)** Statistical analysis of the proportions of tumor area as represented in **(A–F)** (*n* = 5). Data are presented as mean ± SD. Ns, non-significant; *p < 0.05; **p < 0.01.

To verify whether IFNγ or CCL2 plays a role in YB1-mediated antitumor process, we gave neutralizing antibodies against IFNγ and/or CCL2 twice a week after tail vein injection of YB1. The results showed that the neutralizing antibody of CCL2 alone could not weaken the antitumor effect of YB1, but in the neutralizing antibody group of IFNγ or IFNγ + CCL2, the antitumor effect of YB1 was significantly weakened (Figures 3A–G). These results suggested that the upregulation of IFNγ in the liver is indispensable for the anti-tumor effect of YB1 therapy.

### AAV-IFNr completely inhibit INR1G9 tumor growth in the liver

**Figure 4:**
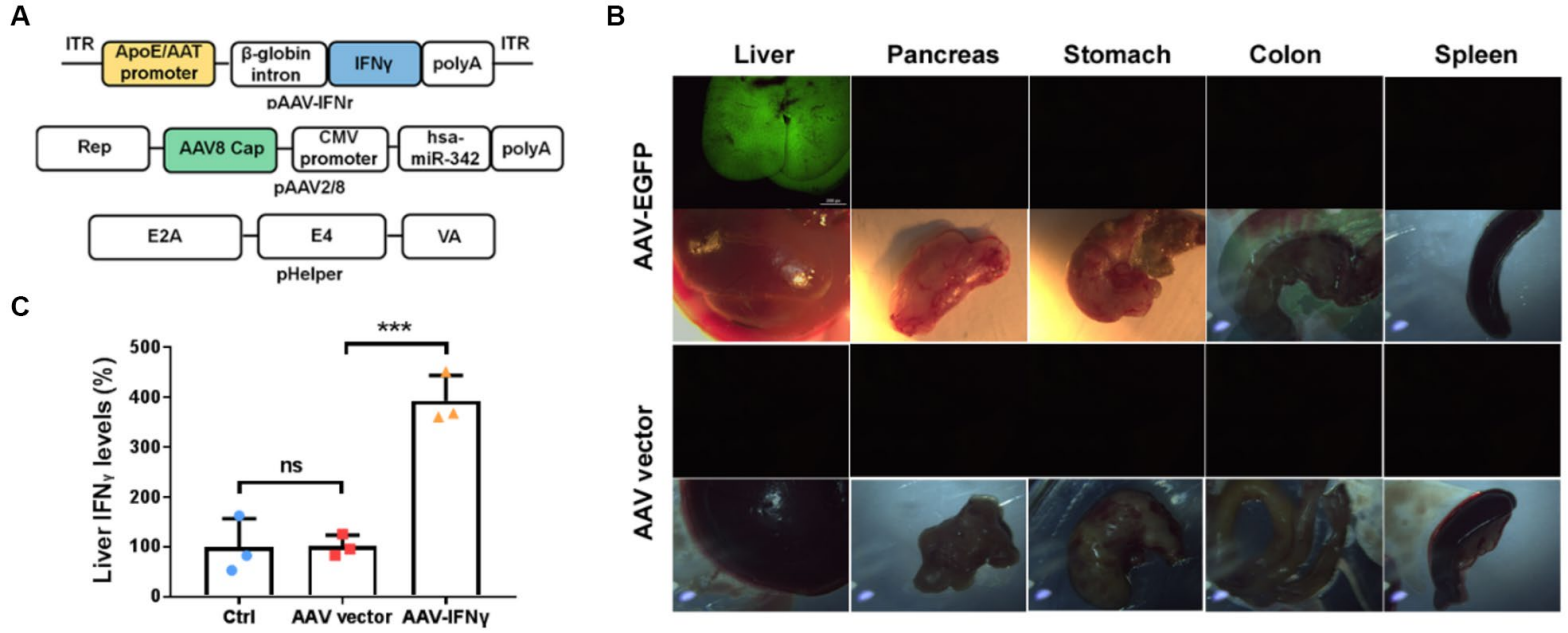
Liver-targeted expression based on AAV2/8 vectors. **(A)** Schematical diagrams of liver-targeted AAV2/8 vector containing the IFNγ-expressing cassette and packaging plasmids. **(B)** Green fluorescence detected in the liver, pancreas, stomach, colon and spleen from mice injected with AAV-EGFP or AAV vector for one week. **(C)** Relative IFNγ levels measured by ELISA in the livers of mice injected with PBS, AAV vector or AAV-IFNr (*n* = 3). Data are presented as mean ± SD. **p < 0.01.

To verify that whether liver-targeted IFNγ has a similar anti-tumor effect as YB1, we prepared chimeric AAV2/8 specifically targeting the liver as an IFNγ delivery vector (Figure 4A). At the same time, AAV2/8 expressing EGFP (AAV-GFP) was prepared to observe the specific targeting of the virus vector to the liver. We found that one week after tail vein injection of AAV-GFP, there was significant green fluorescence in the liver, but not in other organs of mice (Figure 4B). We also detected IFNγ in the liver of AAV-IFNr infected mice two weeks post injection. It was found that IFNγ level was significantly higher in the liver of mice infected with AAV-IFNr than that of the negative control group (Figure 4C).

**Figure 5:**
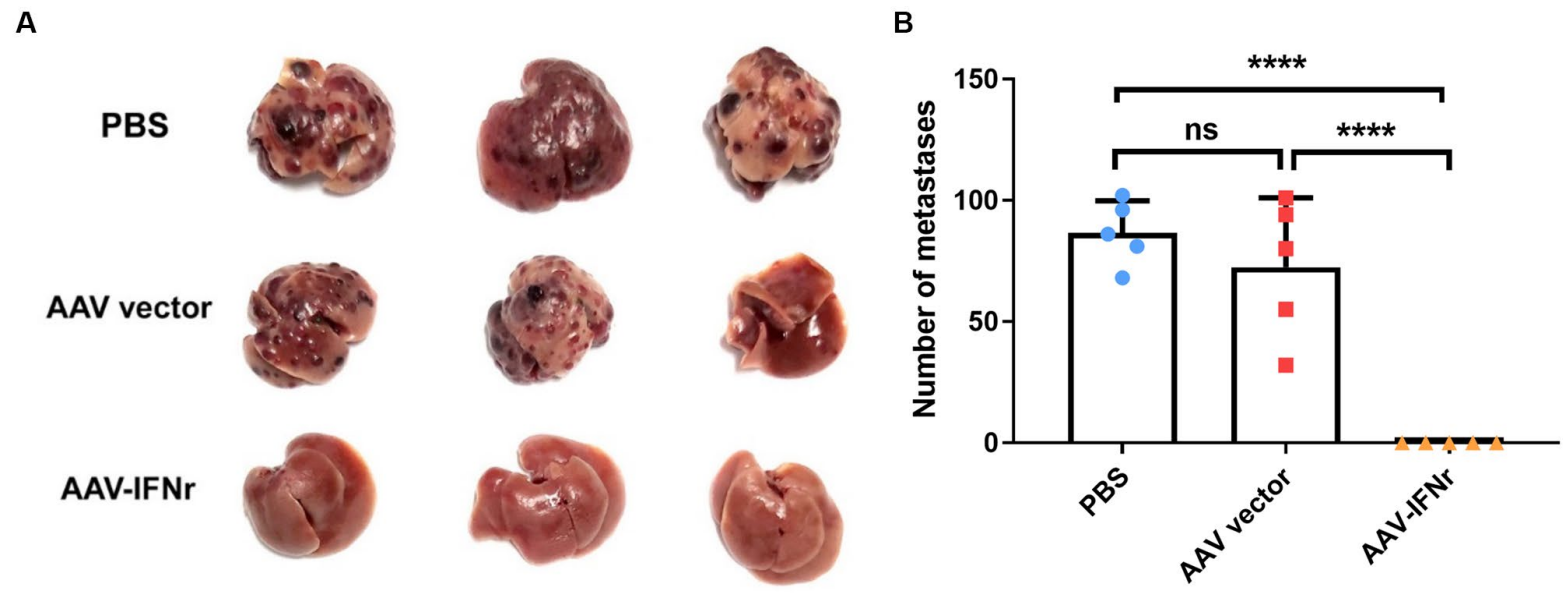
AAV-IFNr completely inhibited the liver metastases of INR1G9 cells. **(A)** Representative pictures of the livers from mice inoculated with INR1G9 cells in the spleen and injected with PBS, AAV vector or AAV-IFNr through the tail veins (*n* = 5). **(B)** Statistical analysis of the number of liver metastases in the PBS, AAV vector and AAV-IFNr groups. Data are presented as mean ± SD. ****p < 0.0001.

We evaluated the effect of AAV-IFNr on liver tumorigenesis. The injection of AAV empty vector alone could not have a significant effect on tumor formation. However, there was no visible tumor formation in the liver of mice injected with AAV-IFNr (Figure 5). These results proved that the liver-targeted IFNγ expression has a strong ability to inhibit tumor formation in the liver.

### Macrophages and NK cells are activated upon AAV-IFNr treatment

**Figure 6:**
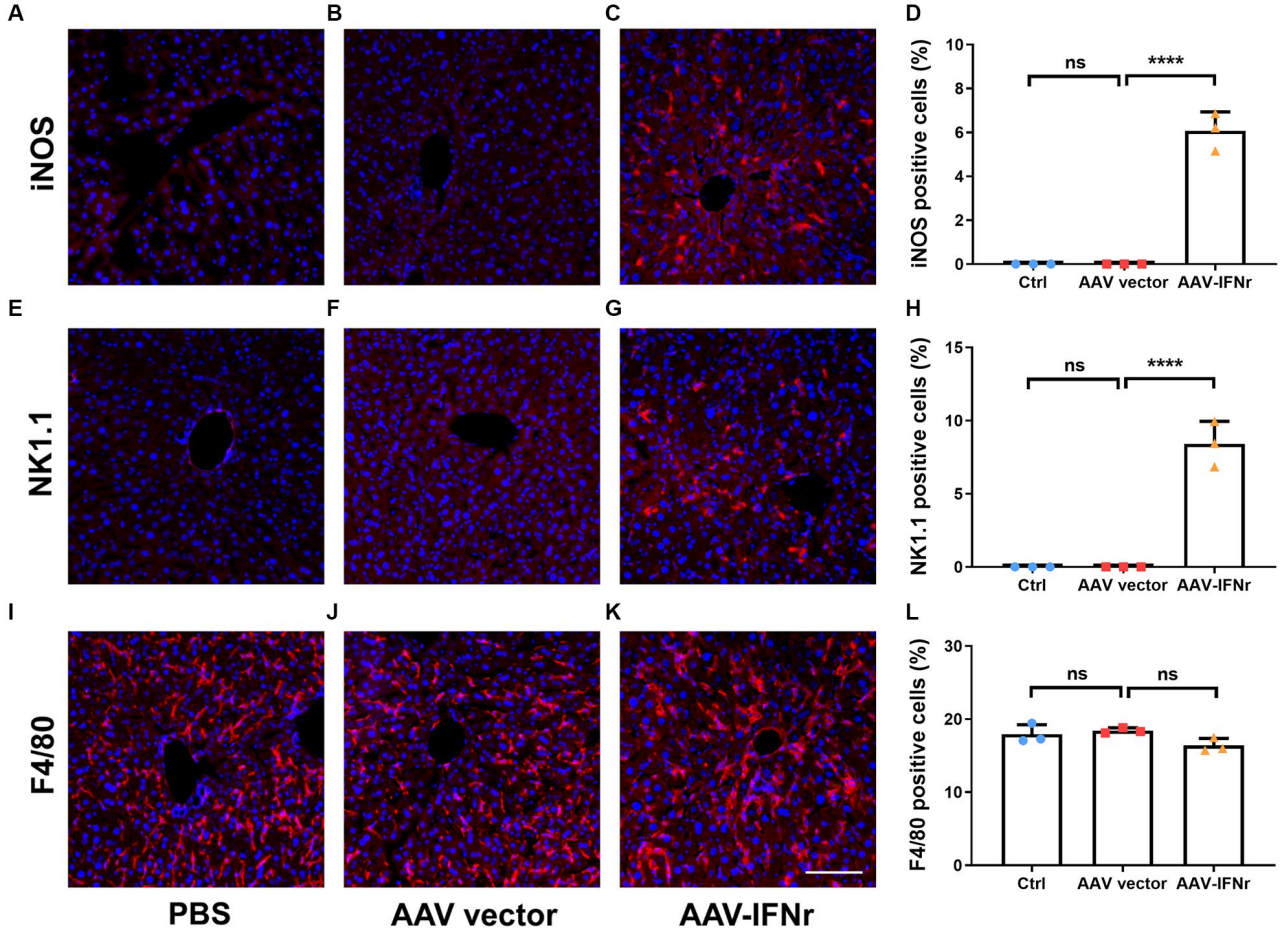
AAV-IFNr activated macrophages and NK cells in the liver. Detection of iNOS **(A–C)**, NK1.1 **(E–G)** or F4/80 **(I–K)** by immunofluorescence in the liver of mice (*n* = 3) injected with PBS **(A,E,I)**, AAV vector **(B,F,J)** or AAV-IFNr **(C,G,K)** through tail vein for two weeks. Scale bar: 100 μm. The statistical analyses were presented as mean ± SD for iNOS **(D)**, NK1.1 **(H)** and F4/80 **(L)** as indicated. ****p < 0.0001. ns, non-significant.

We examined the activation of macrophages and NK cells in the liver after AAV-IFNr treatment. We found that no obvious activated M1 macrophage or NK cells were present in the PBS or empty vector group, there were significant number of cells stained positive for the activation marker iNOS for M1 macrophages (Figures 6A–D) and NK1.1 for NK cells (Figures 6E–H). The percentages of F4/80 positive cells representing for total macrophages showed no significant difference among these groups (Figures 6I–L). These data indicate that the expression of IFNγ promoted the M1 polarization of macrophages and the activation of NK cells, which may strengthen the IFNγ-mediated antitumor effect.

## Discussion

Immunotherapy, especially anti-CTLA-4/PD-1/PD-L1 antibodies, has been applied in multiple types of cancers. However, the tumor mutation burden, PD-L1 expression and T cell infiltrations are quite heterogeneous among tumors, which may account for the inconsistent treatment effect (17–19). For neuroendocrine tumors, clinical trials with immunotherapy as monotherapy or combinations have shown limited efficacy (20). Therefore, development of new therapy for this kind of tumor is of great importance. Activation of the innate immunity maybe one of the choices.

The genetically engineered salmonella strain YB1 or YB1-derived vectors has been reported to treat multiple types of tumors including breast cancer, hepatic cancer, neuroblastoma, and lung or liver metastases (9–12). Except for the extra anti-tumor agent incorporated in the YB1-derived vectors, the YB1 itself can efficiently stimulate the innate immunity to suppress tumor growth. In a lung metastasis model, YB1-induced IFNγ expression was found to be responsible for its anti-tumor effect (12). In the present study, we verified the role of IFNγ in a YB1-treated liver metastasis model.

The antitumor effect of IFNγ has been identified for a long time. Binding of IFNγ to its receptor can stimulate JAK/STAT1 pathway and induce the expression of transcription factor IRF1, which further activates the downstream target gene expression (21). IFNγ receptor genes IFNGR1, IFNGR2 and the downstream effector IRF1 have been reported to be expressed on a PNET cell line QGP-1, and IFNγ treatment could inhibit tumor growth and induce apoptosis *in vitro*, indicating a direct anti-PNET effect of IFNγ (22).

Other studies suggest that IFNγ mainly plays a role by activating adaptive immunity including upregulating of MHCI expression in professional antigen presenting cells (23), recruiting cytotoxic T lymphocytes (24), promoting Th1 differentiation (25) and driving fragility of regulatory T cells (26). IFNγ pathway is also important for the anti-CTLA-4 therapy (27). However, we used thymus aplastic nu/nu mice for the tumor model in our study, which indirectly suggested that the adaptive immunity can be missing for the anti-tumor effect of YB1 and IFNγ in our model.

It has been demonstrated that IFNγ functions by activating NK cells for the YB1-mediated lung metastasis therapy. IFNγ is also a key cytokine to fully activate macrophages (28). In addition to NK cells, we found that macrophages were also activated to M1 phenotypes by YB1 or AAV-IFNγ therapy. Therefore, we suggested that besides its direct anti-PNET role, IFNγ may also function through activating NK cells and macrophages to exhibit further antitumor effect. Further study is important to understand the role of macrophages in YB1 antitumor effect.

Besides antitumor effect, IFNγ has also been found to have a pro-tumorigenic effect in certain circumstance (29). Injection of recombinant IFNγ was tried in several anti-tumor clinical trials including chronic myelogenous leukemia (30), bladder carcinoma (31), ovarian cancer (32), adult T cell leukemia (29) and melanoma (33), but the results were mixed. Although IFNγ plays a crucial role in YB1-mediated suppression of lung metastasis, intravenous injection of recombinant IFNγ failed to inhibit lung metastasis (12). A possible explanation may be that systematically administration of IFNγ might not be able to achieve a desired concentration in the targeted organs. To circumvent this problem, we engineered targeted IFNγ expression vector based on the chimeric AAV2/8 which harbors the AAV2 backbone and the AAV8 capsid conferring liver-specific targeting. AAV8 targets hepatocytes with high efficiency around 90–95% (34). AAV2/8 mediated FIX delivery to the liver showed safety and efficacy for hemophilia B patients (35). The reconstructed ApoE-AAT promoter further guaranteed liver-specific expression (15). The IFNγ level in the liver significantly elevated two weeks after treatment. Surprisingly, this liver targeted AAV-IFNγ expression vector reproduced an even stronger antitumor efficacy than YB1. Although the splenomegaly was observed as a side effect, the non-permanent expression of AAV vectors may limit this side effect in long term. Therefore, compared with systematically injection of IFNγ, targeted-IFNγ supplement may be more effective for tumor treatment. Our study suggested that liver-targeted IFNγ expression holds great potential in PNETs liver metastasis. We also speculate that targeted IFNγ therapy may also be applied in other organs, which is worthy of further exploration.

## Author’s note

The statistical methods of this study were reviewed by Xiaoying Gu from Biostatistic Service from the Project and Data Management Department, Institute of Clinical Medical Sciences, China-Japan Friendship Hospital.

## Data availability statement

The raw data supporting the conclusions of this article will be made available by the authors, without undue reservation.

## Ethics statement

The animal study was approved by Animal Ethics Committee of China–Japan Friendship Hospital. The study was conducted in accordance with the local legislation and institutional requirements.

## Author contributions

ZH: Writing – review & editing, Writing – original draft, Conceptualization, Investigation. SW: Writing – original draft, Data curation, Investigation, Writing – review & editing. YZ: Writing – original draft, Investigation. XW: Writing – original draft, Data curation. JC: Writing – original draft, Data curation. YL: Writing – original draft, Investigation. CY: Writing – original draft, Investigation. MZ: Writing – original draft, Data curation. BD: Writing – review & editing. BY: Writing – original draft, Data curation, Resources. J-DH: Writing – original draft, Data curation, Resources. ZW: Writing – original draft, Conceptualization, Data curation, Investigation, Supervision, Writing – review & editing. JZ: Writing – original draft, Conceptualization, Supervision, Writing – review & editing.

## Footnotes

^1^ https://www.ncbi.nlm.nih.gov/nuccore/af513852

## Supplementary material

Supplementary Figure S1 Salmonella cannot effectively infect INR1G9 cells. INR1G9 or Huh7.5 cells were incubated with 5 × 10^6^ YB1 for 24 hours. Cells were fixed, permeabilized and immunostained with anti-salmonella antibody (green). Scale bar: 25 μm.
