# Supplementary figures and images for: Targeted IFNγ induction by a genetically engineered Salmonella typhimurium is the key to the liver metastasis inhibition in a mouse model of pancreatic neuroendocrine tumor
Source: Front Med (Lausanne). 2023 Oct 31;10:1284120. doi: 10.3389/fmed.2023.1284120 (PMC10644712; doi:10.3389/fmed.2023.1284120)

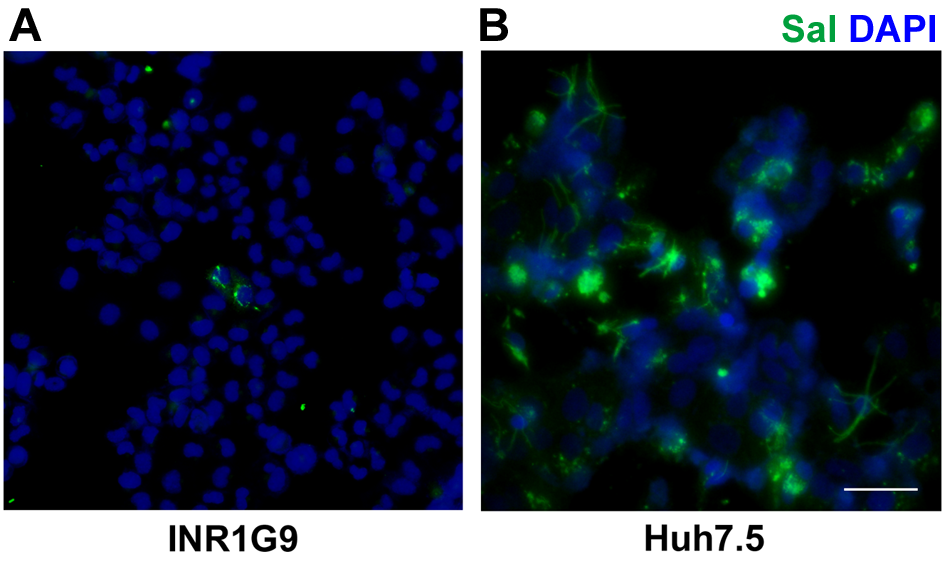

Supplement: Supplementary Figure S1 — Salmonella cannot effectively infect INR1G9 cells. INR1G9 or Huh7.5 cells were incubated with 5 × 10^6^ YB1 for 24 hours. Cells were fixed, permeabilized and immunostained with anti-salmonella antibody (green). Scale bar: 25 μm. [file Image_1.TIF]
